# Therapeutic management of axial spondyloarthritis: summary of 2024 update of the Spanish clinical practice guideline (ESPOGUIA)

**DOI:** 10.1177/1759720X261464260

**Published:** 2026-08-01

**Authors:** Clementina López-Medina, Carlos Montilla-Morales, Mireia Moreno, Manuel Jose Moreno-Ramos, Rubén Queiro, Julio Ramírez, David Díaz-Valle, Agnès Fernández-Clotet, Josep Riera, Petra Díaz-del Campo, Juan D. Cañete, Victoria Navarro-Compán

**Affiliations:** Hospital Reina Sofía, IMIBIC, University of Córdoba, Córdoba 14004, Spain; Faculty of Medicine of the University of Salamanca, Salamanca, Spain; Parc Taulí Research and Innovation Institute (I3PT), Hospital Parc Taulí and Universidad Autónoma de Barcelona, Barcelona, Spain; Hospital Virgen de la Arrixaca, Biomedical Research Institute of Murcia Pascual Parrilla–IMIB, Murcia, Spain; Rheumatology and ISPA Translational Immunology Division, Central University Hospital of Asturias, Oviedo University School of Medicine, Oviedo, Spain; Hospital Clínic and IDIBAPS, Barcelona, Spain; Hospital Clínico San Carlos, Madrid, Spain; Hospital Clínic, Barcelona, Spain; Hospital Clínic, Barcelona, Spain; Spanish Society of Rheumatology (SER), Salamanca, Madrid, Spain; Rheumatology Department, Hospital Clinic and IDIBAPS, Barcelona, Spain; Hospital Universitario La Paz, IdiPaz, Madrid, Spain

**Keywords:** axial spondyloarthritis, clinical practice guideline, evidence-based recommendations, multidisciplinary management

## Abstract

Axial spondyloarthritis (axSpA) is a chronic inflammatory rheumatic disease associated with significant morbidity and impaired quality of life. Rapid advances in diagnosis, monitoring, and especially treatment over the past decade have increased the complexity of axSpA management and contributed to variability in clinical practice. To address this need, the Spanish Society of Rheumatology (SER) has developed an updated version of ESPOGUÍA, its clinical practice guideline for spondyloarthritis. This review summarizes the updated recommendations. A multidisciplinary panel (including rheumatologists, other medical specialists, nurses, physiotherapists, methodologists, and patients) formulated key clinical questions using the PICO framework. Systematic literature reviews covering the period 2018–2023 were conducted across major databases. Evidence quality and strength of recommendations were assessed using the Grading of Recommendations Assessment, Development, and Evaluation (GRADE) methodology. When evidence was insufficient, recommendations were formulated through a structured deliberative consensus process using the GRADE Evidence-to-Decision framework. Patient representatives actively contributed throughout the process, and the draft guideline underwent public consultation. ESPOGUÍA provides evidence-based recommendations for the management of adult patients with axSpA, focusing on pharmacological and non-pharmacological interventions. The guideline addresses the use of biologic and targeted synthetic disease-modifying antirheumatic drugs, prognostic factors for treatment response and radiographic progression, management of treatment failure, and strategies for treatment optimization, including dose tapering. Special attention is given to extra-musculoskeletal manifestations such as uveitis, inflammatory bowel disease, and psoriasis. Non-pharmacological approaches—including physical activity, smoking cessation, weight management, and nurse-led education—are emphasized as integral components of comprehensive care. ESPOGUÍA 2024 offers a comprehensive, evidence-based framework to support harmonized and patient-centered management of axSpA in Spain. By integrating recent therapeutic advances and multidisciplinary perspectives, the guideline aims to reduce variability in clinical practice, improve outcomes, and enhance quality of life for patients with axSpA, while also identifying key areas for future research.

## Introduction

Axial spondyloarthritis (axSpA) is a chronic inflammatory rheumatic disease that mainly affects the axial skeleton, although peripheral and extra-musculoskeletal manifestations can also appear.^1^ The main clinical symptom is chronic back pain, reflecting inflammation in the spine, which can lead to ankylosis and a decrement of mobility in late stages of the disease.^1–3^

In a relatively short period of time, remarkable progress has been made in many aspects of axSpA management, including diagnosis and monitoring, but especially treatment. This growing complexity, together with these rapid advances, has led to some degree of variability in clinical practice. This highlights the need for a clinical practice guideline for axSpA. To reduce variability in clinical practice and improve outcomes for patients with axSpA, the Spanish Society of Rheumatology (SER) has led the development and update of ESPOGUÍA, its clinical practice guideline (CPG).^4^ This guideline builds upon previous editions published in 2009, 2015, and 2018. ESPOGUÍA provides a structured framework of evidence-based recommendations aimed at optimizing patient care through systematic literature reviews and risk–benefit evaluations of available therapeutic options.^4^ ESPOGUÍA includes clinical guidance for both axSpA and psoriatic arthritis (PsA), but in this manuscript, only those points related to axSpA will be addressed.

The main purpose of ESPOGUÍA is to help rheumatologists and other clinicians make treatment decisions for adults with axSpA based on the best available evidence.^3,4^ For this reason, this document was created by a multidisciplinary team of healthcare professionals from across Spain who are involved in caring for patients with axSpA.^4^

The guideline’s specific aims include strengthening the clinical skills of healthcare professionals involved in the care of people with axSpA, reducing variability in treatment decisions, and critically evaluating the efficacy, safety, and cost-effectiveness of both pharmacological and non-pharmacological therapies. It also seeks to summarize and disseminate the best available scientific evidence to enhance professional knowledge and ultimately improve patients’ quality of life. Additional objectives include providing clear guidance to standardize axSpA management, promoting effective collaboration among key specialties (particularly rheumatology, dermatology, ophthalmology, and gastroenterology), and offering accessible educational material to patients, their families, and caregivers to support a better understanding of the disease and its course.^3,4^

Major therapeutic progress since the 2018 edition motivated the development of this updated guideline. Among the most relevant advances are the introduction of new biologic options for axSpA, including IL-17A/F inhibitors and JAK inhibitors (JAK1, JAK3).^5,6^ This update also responds to key clinical questions, such as whether biological and targeted synthetic disease-modifying antirheumatic drugs (b/tsDMARDs) slow radiographic progression, which factors predict clinical response to IL-17 or JAK inhibition, the effectiveness of b/tsDMARDs in extra-musculoskeletal manifestations, and whether dose tapering or treatment discontinuation is advisable in patients with stable disease.

The intended audience extends beyond rheumatologists, encompassing dermatologists, gastroenterologists, ophthalmologists, physiotherapists, nurses, primary care physicians, and patients themselves. Ultimately, the guideline promotes coordinated, integrated, and patient-centered care grounded in the latest scientific evidence.

## Methods

The ESPOGUÍA CPG was created using a systematic, rigorous approach designed to guarantee the robustness and clinical relevance of its content. Coordinated by the SER, the process followed internationally recognized standards for guideline development.^7–10^ A multidisciplinary team was assembled through an open call launched by the SER, bringing together rheumatologists, dermatologists, gastroenterologists, ophthalmologists, primary care physicians, physiotherapists, nurses, patients, and methodologists. Members were selected for their clinical expertise, research experience, and direct involvement in managing spondyloarthritis, ensuring that the final document captured the full complexity of these diseases.

To structure the evidence, review it, and guide the formulation of recommendations, the working group developed clinical questions using the PICO format (Population, Intervention, Comparison, Outcome; Supplemental Table 1). This framework helped identify the most relevant issues related to diagnosis, treatment, and patient care, and directed the assessment of both pharmacological and non-pharmacological strategies.^7–9^

### Literature search

A comprehensive systematic review of the scientific literature from 2018 to 2023 was conducted for each PICO question. Searches were performed by an expert librarian in major databases—Medline (via PubMed), Embase (Elsevier), Cochrane Library including CENTRAL (Wiley Online Library), and CINAHL (EBSCOhost) —to retrieve randomized trials, observational studies, and systematic reviews, with an emphasis on recent evidence applicable to the Spanish clinical setting. For questions carried forward from the previous ESPOGUÍA 2015/2018 updates, the original search strategies were updated and limited to studies published after the searches performed for those earlier versions, that is, from 2015 or 2016 onwards, depending on the topic. For newly developed questions, no date restriction was applied. Searches were closed in August 2023. When the electronic search retrieved few or non-informative studies, additional manual searches of reference lists from relevant articles were undertaken. References proposed by panel members and external reviewers were also considered, allowing the inclusion of selected relevant studies published in 2024 before completion of the final guideline. Studies published in English, Spanish, and French were eligible. References were managed using EndNote X7. When high-quality evidence was unavailable, the group considered indirect or lower-level data, clearly acknowledging their limitations.^7–9^ More detailed information on the literature search is provided in the original full version of ESPOGUIA.^11^

Because ESPOGUÍA 2024 was a partial update of previous editions of the guideline, there was no single study-selection process for the whole document. Different types of clinical questions were addressed: questions from previous ESPOGUÍA versions that remained valid and did not require updating; questions for which a narrative update was performed, prioritizing systematic reviews and clinical practice guidelines; new PICO questions requiring full systematic literature reviews; and questions that did not require reformulation in PICO format and were addressed through narrative evidence review. Therefore, PRISMA flow diagrams were generated for the individual PICO questions for which systematic literature reviews were conducted, rather than as a single global PRISMA diagram for the entire guideline. The complete search strategies and the corresponding PICO-specific PRISMA flow diagrams are provided in the Methodological Appendix from the original document (pages 26, 32, 38, and 42).^12^

- Study inclusion criteria: Eligible studies included adults with radiographic (r-axSpA) or non-radiographic axSpA (nr-axSpA), and interventions such as early therapeutic approaches, conventional synthetic DMARDs (csDMARDs), b/tsDMARDs, predictors of treatment response and structural progression, treatment tapering or discontinuation, treatment failure, smoking, obesity, and patient education initiatives, including other health-related lifestyle factors. Outcomes assessed included clinical measures of disease activity, peripheral and axial symptoms, radiographic progression, inflammatory biomarkers, flare rates, dactylitis, enthesitis, uveitis, psoriasis, inflammatory bowel disease, and treatment response. Study designs considered for inclusion encompassed systematic reviews of randomized controlled trials, phase III–IV double-blind RCTs, trial sub-analyses, and observational studies.
- Studies exclusion criteria: Studies in pediatric populations, adolescents, or pregnant women were excluded, as were studies not aligned with the corresponding PICO question in terms of population, intervention, comparator, outcomes, or design. Abstracts, posters, narrative reviews, letters, editorials, and unpublished studies were also excluded.

### Analysis and synthesis of the scientific evidence

The quality of the evidence and the strength of each recommendation were evaluated using the Grading of Recommendations Assessment, Development, and Evaluation (GRADE) methodology. In addition to study design and methodological quality of individual studies, GRADE considers other factors that influence certainty in the estimates, including consistency of results across studies, directness or indirectness of the evidence, precision of the estimates, and risk of publication bias.^7–9^ Based on the overall assessment of these domains, the certainty of the evidence for each critical or important outcome was rated as high, moderate, low, or very low. Recommendation strength was then determined by considering not only the certainty of the evidence, but also the balance between desirable and undesirable effects, patients’ values and preferences, feasibility, resource implications, and clinical applicability. Accordingly, in some cases, strong recommendations could be issued despite low or moderate certainty of evidence when the panel considered that the expected benefits clearly outweighed potential harms or when the statement reflected widely accepted standards of care. Recommendations were formulated by the guideline development group using the GRADE Evidence-to-Decision framework and the Clinical Practice Guidelines and Recommendations of the SER.^13^ This framework considered the certainty of the evidence, the balance between desirable and undesirable effects, patient values and preferences, feasibility, equity, acceptability, resource implications, and other relevant contextual factors. For each clinical question, evidence summaries and Evidence-to-Decision frameworks were discussed by the multidisciplinary panel. A structured consensus process was undertaken during face-to-face meetings of the task force. Proposed statements were reviewed, discussed, and revised through iterative rounds of deliberation. Consensus was considered achieved when at least 70% of task force members agreed with the final wording of each statement. Good clinical practice statements were reserved for situations in which formal evidence was limited or unavailable, but the panel considered the recommendation essential for appropriate clinical practice.

### Role of the patients’ research partners

Patients played an essential role in the development of the guideline. Their involvement ensured that the recommendations reflected meaningful outcomes, everyday challenges, and priorities related to quality of life and communication in clinical care.^10^

In the final stage, the draft guideline was opened for public consultation, allowing professionals, patients, and other interested parties to provide feedback. This step ensured transparency, encouraged external input, and strengthened consensus prior to publication of the final version.^9^

Two face-to-face meetings were held for the multidisciplinary team: one for deciding the PICO questions, and a second to discuss and reach consensus on the different points of this guideline.

## Clinical topics addressed in ESPOGUIA related to axSpA (Table 1)

### Treatment with biological DMARDs or JAK inhibitors versus placebo

Recent advances in the management of axSpA have expanded therapeutic options beyond TNF inhibitors (TNFi; i.e., adalimumab, infliximab, etanercept, golimumab, and certolizumab pegol) to include IL-17 inhibitors (IL-17i; i.e., secukinumab, ixekizumab, and bimekizumab) and JAK inhibitors (JAKi; i.e., tofacitinib and upadacitinib). These newer therapies have demonstrated significant efficacy and acceptable safety profiles in randomized, double-blind, placebo-controlled trials involving both r-axSpA and nr-axSpA, with acceptable short-term safety in the trial setting. However, treatment selection should also take into account post-authorization safety information, particularly for JAK inhibitors.^5,6,14–16^ As a result, their use is recommended in patients with active disease who have not responded to or cannot tolerate NSAIDs. The choice between these drug classes should be individualized, taking into account each patient’s clinical characteristics, comorbidities, and risk factors.

**Table 1.** ESPOGUIA recommendations for axial spondyloarthritis.

**Recommendation 1:** In patients with active axSpA, the use of IL-17A inhibitors, IL-17A/F inhibitors, and JAK inhibitors is recommended as one of the therapeutic options after failure and/or intolerance to NSAIDs. The choice of treatment line will depend on the patient’s clinical characteristics. (Strong recommendation)
**Recommendation 2:** The guideline development group considers the available evidence to be insufficient and/or inadequate to issue a definitive recommendation on the effect of biological DMARDs or JAK inhibitors in slowing structural damage progression in patients with axSpA; however, it is suggested that predictive factors of structural progression be taken into account when considering these treatments. (Good clinical practice)
**Recommendation 3:** In patients with active axSpA who are about to start treatment with IL-17A or IL-17A/F inhibitors, it is suggested to identify predictors of good therapeutic response, such as male sex and elevated C-reactive protein (CRP). (Weak recommendation)
**Recommendation 4:** In patients with active axSpA who are about to start treatment with IL-17A or IL-17A/F inhibitors, it is suggested to identify predictors of radiographic progression, such as male sex, older age, smoking, elevated C-reactive protein (CRP), HLA-B27 positivity, and bone marrow edema on spinal MRI. (Weak recommendation)
**Recommendation 5:** In patients with axSpA who do not respond to a first TNF inhibitor, it is recommended to use another TNF inhibitor, an IL-17A or IL-17A/F inhibitor, or a JAK inhibitor. (Strong recommendation)
**Recommendation 6:** In patients with axSpA who have maintained low disease activity or remission for at least six months, it is recommended to consider reducing the frequency of biological DMARD administration, in agreement with the patient and with ongoing clinical monitoring. (Strong recommendation)
**Recommendation 7:** In patients with axSpA who have sustained low disease activity or remission, the systematic discontinuation of biological DMARD treatment is not recommended due to the increased risk of disease flare. (Strong recommendation)
**Recommendation 8:** In patients with axSpA and uveitis, the use of monoclonal TNF inhibitors is recommended for the prevention of anterior uveitis episodes. (Strong recommendation)
**Recommendation 8.1:** TNF inhibitors, particularly adalimumab, are also recommended for the treatment of refractory or recurrent anterior uveitis when conventional therapies have failed. (Good clinical practice)
**Recommendation 8.2:** In patients with axSpA, the use of etanercept is not recommended for either the prevention or treatment of anterior uveitis episodes. (Good clinical practice)
**Recommendation 9:** In patients with axSpA, the guideline development group considers that there is no evidence to recommend the use of IL-17 inhibitors or JAK inhibitors for the prevention or treatment of anterior uveitis episodes. (Good clinical practice)
**Recommendation 10:** In patients with axSpA and active inflammatory bowel disease, the use of monoclonal TNF inhibitors and JAK inhibitors is recommended for managing intestinal inflammation. (Strong recommendation)
**Recommendation 11:** In patients with axSpA and inflammatory bowel disease, the use of IL-17 inhibitors is not recommended. (Strong recommendations against)
**Recommendations 12:** Because psoriasis is less common in axSpA, there is less evidence regarding the effectiveness of different treatments for psoriasis; therefore, it is suggested to refer to the recommendations established for psoriatic arthritis. (Good clinical practice)
**Recommendation 13:** In adult patients diagnosed with axSpA, it is suggested to incorporate exercise programs as part of disease management to improve symptoms, disease activity, quality of life, and health-related physical fitness. (Weak recommendation)
**Recommendation 14:** These programs should include aerobic exercises and should preferably be performed in a supervised setting by a physiotherapist, in group sessions. (Weak recommendation)
**Recommendation 15:** In patients with axSpA, smoking cessation and maintaining a body mass index (BMI) between 18.5 and 25 are recommended to improve disease control. (Strong recommendation)

axSpA, axial spondyloarthritis.

The recommendations emphasize special considerations for subgroups. For patients with nr-axSpA, objective signs of inflammation—such as elevated C-reactive protein or MRI evidence—should be confirmed before initiating these therapies. Although differences exist between individual IL-17i (IL-17A vs IL-17A/F) and among JAKi, current evidence does not allow conclusions about superiority within each class; therefore, recommendations are made at the drug-class level. With regard to safety prevention, special caution is required when considering JAK inhibitors. In line with EMA/PRAC recommendations, JAK inhibitors should generally be used only if no suitable treatment alternatives are available in patients aged 65 years or older, in current or past long-term smokers, and in those with established atherosclerotic cardiovascular disease, other major cardiovascular risk factors, or malignancy risk factors. In addition, JAK inhibitors should be used with caution in patients with other risk factors for venous thromboembolism, such as previous VTE, major surgery, immobilization, use of combined hormonal contraceptives or hormone replacement therapy, or inherited coagulation disorders, and these risks should be reassessed periodically during treatment. Patients should also be screened for tuberculosis before treatment initiation and monitored closely for serious infections during therapy.

Overall, IL-17i and JAKi are considered effective, acceptable, and feasible treatment options within clinical practice. Access inequities are not expected in the current healthcare context, and the long-standing experience of rheumatologists with advanced therapies supports their implementation. While cost analyses were not performed, the guideline group noted insufficient information to comment on resource use. Patients are also expected to value the key outcomes consistently across studies, reinforcing the clinical relevance of these recommendations.

### Prognostic factors of radiographic progression and treatment response

Current evidence exploring whether biological DMARDs or JAKi can prevent structural progression in axSpA remains inconclusive.^17–21^ Although these therapies consistently reduce inflammation in the sacroiliac joints and spine, the available data—particularly for the newest drugs, JAKi— are largely derived from pivotal clinical trials, and no clear therapeutic “window of opportunity” has been established.^22^ While TNFi, as well as IL-17i, have shown low rates of radiographic progression over 2 years, head-to-head comparisons such as the SURPASS trial indicate similar effects between TNFi and IL-17i.^21^ Studies combining biologics with NSAIDs demonstrate improved clinical and imaging outcomes but have not yet clarified their impact on long-term structural progression.^23–25^

Given these uncertainties, the guideline panel recommends focusing on prognostic factors of structural damage when selecting therapy. Predictors of structural damage progression include baseline radiographic abnormalities, elevated inflammatory markers (such as CRP), MRI evidence of active inflammation at the sacroiliac joints and/or spine, male sex, smoking, older age, and HLA-B27 positivity. In clinical practice, the presence of several of these factors may identify patients at higher risk of progression, in whom delays in starting advanced therapy should be strongly avoided. For example, in a patient with elevated CRP, active spinal and/or sacroiliac MRI lesions, smoking, and baseline syndesmophytes, the clinician may favor earlier initiation of a b/tsDMARD rather than prolonging more conservative treatment strategies. By contrast, in a patient without objective signs of inflammation or structural damage, a standardized stepwise approach may be reasonable.

However, these prognostic factors should not currently be interpreted as favoring one specific mechanism of action over another, since available evidence does not demonstrate that TNFi, IL-17i, or JAKi differ clearly in their ability to prevent structural progression according to these profiles. Therefore, once the need for advanced therapy has been established, the choice of mechanism of action should still be guided primarily by the overall clinical phenotype, extra-musculoskeletal manifestations, comorbidities, safety profile, and patient preferences. Similarly, in relation to treatment adaptation or tapering, the presence of poor prognostic factors may support a more cautious approach in patients at high risk of progression.

Overall, the guideline supports incorporating prognostic factors into therapeutic decision-making to better adapt treatment strategies for patients with axSpA. However, recommendations remain weak because of reliance on observational studies and the limited robustness of the available evidence.

### Treatment failure

**Figure 1.**
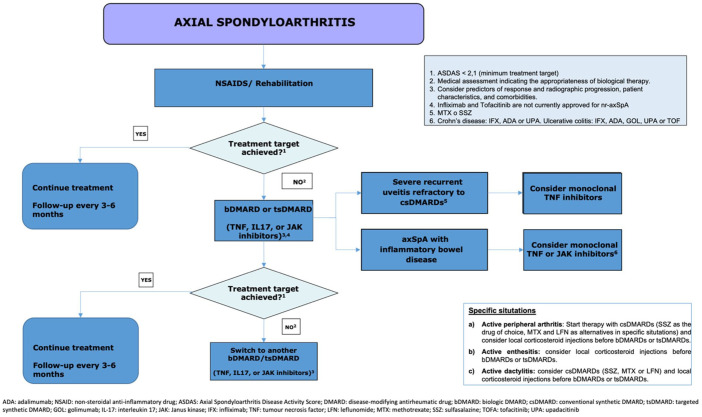
Treatment algorithm for axial spondyloarthritis. ADA, adalimumab; ASDAS, Axial Spondyloarthritis Disease Activity Score; bDMARD, biologic DMARD; csDMARD, conventional synthetic DMARD; DMARD, disease-modifying antirheumatic drug; GOL, golimumab; IFX, infliximab; IL-17, interleukin 17; JAK, Janus kinase; LFN, leflunomide; MTX, methotrexate; NSAID, non-steroidal anti-inflammatory drug; SSZ, sulfasalazine; TNF, tumor necrosis factor; TOFA, tofacitinib; tsDMARD, targeted synthetic DMARD; UPA, upadacitinib.

In patients with axSpA who experience treatment failure with a first TNFi (whether due to loss of efficacy or adverse events), the guideline strongly recommends switching to another TNFi, an IL-17i, or a JAKI. Evidence from clinical trials and observational cohorts shows that changing to a different therapeutic target or cycling within the same class can provide meaningful clinical benefit, with no significant differences observed between TNFi, IL-17i, and JAKi in terms of efficacy or drug retention (Figure 1).^26–30^ Subgroup considerations are important, particularly in patients aged ⩾65 years, active smokers, or those with cardiovascular or cancer risk, where alternatives to JAKi should be prioritized, or the lowest effective dose used when JAKi are necessary.

There is evidence suggesting a better response and survival in patients who switch to a second TNF inhibitor because of secondary failure or toxicity of the first, compared with patients who present with primary lack of response to TNF inhibitors. However, the quality of evidence from the studies identified in the systematic review is very low. Although in this situation of primary failure to respond to a TNF inhibitor, it would be reasonable to consider a change in therapeutic target, given the very low quality of evidence, the working group decided not to include a specific recommendation on this matter. This is also in line with the cautious stance because of the lack of evidence in the ASAS-EULAR international guidelines for the management of axSpA.^3^ In addition, when selecting the next therapeutic agent, clinicians should take into account objective markers of inflammation, the presence or flare occurrence of extra-musculoskeletal manifestations to guide drug choice, and individual safety considerations, including cardiovascular and malignancy risks.

Although real-world data remain limited—especially regarding later treatment lines and sequencing after IL-17i or JAKi failure—available evidence supports the flexibility to switch between therapeutic classes based on individual patient characteristics. Newer agents such as ixekizumab, bimekizumab, tofacitinib, and upadacitinib have demonstrated efficacy in clinical trials involving patients previously exposed to TNF or IL-17 inhibitors, although these findings still require confirmation in routine practice.^5,6,15^ Overall, the accumulated clinical experience suggests that targeted therapies can be sequentially adjusted to optimize treatment response and tolerability in patients with axSpA.

### Treatment optimization

Treatment optimization in axSpA focuses on safely reducing treatment intensity in patients who have achieved sustained low disease activity or remission. Current evidence supports considering a reduction in the frequency of biological DMARD administration—after at least 6 months of stable disease—through shared decision-making and continued clinical monitoring. This strategy, endorsed by strong recommendations, aims to minimize medication exposure while maintaining disease control. In contrast, the systematic discontinuation of biological therapy is not advised, as multiple clinical trials show significantly higher rates of disease flare and loss of disease control when treatment is stopped entirely. This conclusion is supported by randomized controlled trials evaluating treatment withdrawal in both radiographic and non-radiographic axSpA, including studies with adalimumab, certolizumab pegol, ixekizumab, and golimumab, which consistently showed poorer maintenance of remission or low disease activity after complete discontinuation compared with standard maintenance therapy.^31–34^

Studies evaluating treatment tapering strategies demonstrate that while full withdrawal frequently leads to relapse, spacing out doses can maintain remission or low disease activity in many patients. The available evidence includes several randomized trials of dose-spacing strategies, mainly with TNFi, such as etanercept and infliximab-based regimens, as well as studies of progressive interval extension in patients with sustained remission or low disease activity. In addition, trials such as those by Landewé and Weinstein also included interval-extension arms in early axSpA populations with sustained inactive disease. Most of the evidence comes from trials involving TNFi, whereas evidence for IL-17i is limited to withdrawal studies, and no evidence is currently available for JAKi tapering or discontinuation strategies.^35–41^ Despite variability among studies in tapering methods, remission duration before tapering, and follow-up time, results consistently indicate that extending dosing intervals is comparable to continuing standard doses, without compromising disease stability. However, heterogeneity across studies and relatively small sample sizes limit the certainty of these findings.

Practical implementation of tapering depends on the drug formulation: intravenous agents such as infliximab allow dose reduction based on weight, whereas subcutaneous therapies rely on extending injection intervals. This distinction probably explains why most studies have evaluated interval extension rather than actual dose reduction. The evidence base is also heterogeneous with regard to the required duration of remission before tapering, which ranged from approximately 12 weeks in some trials to at least 6 months in most studies, as well as the instruments used to define disease control (e.g., ASDAS or BASDAI). Additional considerations include predictors of poorer success with tapering, such as female sex, HLA-B27 negativity, higher physician global assessment, and elevated CRP levels. Safety data suggest that spacing doses may reduce the risk of adverse events. Overall, the guideline development group supports dose-spacing as a reasonable optimization strategy for stable axSpA, provided decisions are individualized, made collaboratively with the patient, and accompanied by regular clinical follow-up.

### Extra-musculoskeletal manifestations

Monoclonal TNFi (infliximab, adalimumab, golimumab, and certolizumab pegol) are the most effective biologic treatments for preventing and managing anterior uveitis in patients with axSpA.^42^ Evidence shows they significantly reduce the incidence of uveitis compared with placebo, unlike etanercept, which does not provide a protective benefit. IL-17i and JAKi have not demonstrated consistent efficacy for uveitis, with limited or indirect evidence to support their use.^6,42^ Therefore, monoclonal TNFi, especially adalimumab and certolizumab pegol, are recommended for the prevention of anterior uveitis, while other targeted therapies are not advised for this indication.

Monoclonal TNFi are also the preferred option for patients with axSpA and coexisting inflammatory bowel disease, as they effectively control intestinal inflammation and demonstrate similar rates of IBD events to placebo.^43^ Etanercept, however, is less effective for IBD and may be associated with higher rates of exacerbation, making it unsuitable in this setting.^42^ IL-17i are discouraged in patients with known IBD due to reports of worsening disease, despite clinical trials showing low event rates comparable to placebo.^43^ In contrast, JAKi such as upadacitinib and tofacitinib appear safe, with real-world and trial data confirming their efficacy in ulcerative colitis, and for upadacitinib also in Crohn’s disease—supporting their use when axSpA coexists with IBD.^6^

For patients with axSpA, JAKi (upadacitinib and tofacitinib) show psoriasis event rates similar to placebo and align with clinical trial data demonstrating their effectiveness in skin disease, although they are not currently licensed for psoriasis.^6,44^ While their efficacy appears less robust than that of biologics, they remain a reasonable option, particularly when used in collaboration with dermatology for moderate-to-severe cases. Overall, JAKi may be considered in axSpA patients with accompanying psoriasis, but treatment selection should be individualized and guided by shared decision-making.

### Physical activity

Physical activity is considered a core component of non-pharmacological management in axSpA.^45^ Exercise should be recommended from the time of diagnosis and adapted to the stage of disease, mobility limitation, enthesitis involvement, functional status, and the presence of structural damage or ankylosis. Exercise programs—particularly those including aerobic activity—have been shown to improve disease symptoms, functional capacity, mobility, quality of life, and cardiorespiratory fitness.^46^ Although the evidence is somewhat heterogeneous and based mainly on patients with moderate or advanced disease, multiple systematic reviews and meta-analyses demonstrate that structured exercise is more beneficial than no intervention, with moderate evidence supporting improvements in physical function, disease activity, and thoracic expansion.^46^ Supervised group-based exercise appears especially effective for enhancing quality of life, and combining exercise with biological therapy offers additional synergistic benefits.^47^ While no single type of exercise is clearly superior, several modalities have shown benefit, including flexibility exercises, progressive strengthening, aerobic conditioning, walking, stationary cycling, swimming, aquatic therapy, pilates, respiratory exercises, and global postural re-education. In early disease with minimal mobility limitation, general recreational aerobic exercise of similar intensity and duration to that recommended for the general population may be appropriate, whereas more specific spinal mobility, postural, stretching, and strengthening programs may be particularly relevant in patients with more established disease, stiffness or functional limitation. Despite these benefits, the optimal exercise protocol for axSpA remains uncertain, and data on early-stage or ankylosed patients are limited.

In addition, the role of biomechanical load should be considered, particularly in early axSpA or in patients with active enthesitis, as excessive or inappropriate mechanical stress may potentially worsen symptoms or contribute to structural damage in vulnerable sites. Nonetheless, the overarching consensus is that exercise programs should be regular, individualized, and ideally supervised by physiotherapists to ensure proper technique and progression. Supervised physiotherapy and group-based rehabilitation programs may offer additional benefits over unsupervised home exercise for some outcomes, although home-based programs remain valuable when tailored and sustained over time. Rehabilitation modalities such as aquatic therapy, respiratory physiotherapy, and postural re-education may be particularly useful in selected patients depending on symptoms and functional impairment.

For this reason, exercise prescription should consider disease stage, enthesitis involvement, functional status, and the risk of local overload, with adaptation of intensity and type of activity when needed. Aerobic activities such as walking and swimming are practical options frequently recommended in axSpA, as they may help maintain spinal extension, preserve shoulder and hip mobility, and improve overall fitness with relatively low joint impact. Aerobic exercise may additionally reduce inflammatory markers and improve cardiovascular health—an important consideration in this population.^48^ Given that only a minority of patients currently meet recommended exercise levels, fatigue and time limitations should be addressed through education, patient-support networks, and adapted activity plans. Overall, physical activity is recommended as a complementary and essential component of axSpA treatment, contributing meaningfully to long-term disease management and patient well-being.

### Smoking and obesity

Accumulating evidence highlights smoking and excess body weight as important modifiable factors that negatively influence the course of axSpA. Observational studies consistently show that smoking is associated with higher disease activity, poorer response to treatment (particularly to TNFi), and accelerated radiographic progression.^49,50^ Although no direct trials demonstrate improvement after smoking cessation, the weight of epidemiological data and clinical experience strongly supports advising all patients to quit smoking. The guideline group recommends active referral to smoking cessation services whenever possible. Similarly, overweight and obesity have been linked to increased disease activity and reduced therapeutic response across several observational cohorts and systematic reviews, with findings consistent across different BMI categories. Despite the low certainty of evidence, maintaining a BMI between 18.5 and 25 is recommended to optimize disease control.^51,52^ These lifestyle measures were considered strong recommendations by the working group; however, the supporting evidence is not uniform and is based mainly on observational data, indirect evidence, and expert consensus, rather than high-certainty axSpA-specific interventional studies.

The impact of excess weight also extends to pharmacokinetics, which may alter drug concentrations and contribute to reduced efficacy of biologic therapies. Studies comparing normal-weight individuals with those who are overweight or obese demonstrate trends toward worse clinical outcomes and lower likelihood of treatment success, although evidence is limited by methodological heterogeneity and small sample sizes.^53^ Based on clinical experience and convergence with international recommendations on treatment monitoring, the guideline group concludes that both smoking cessation and weight management are essential components of comprehensive axSpA care. Patients should be referred to appropriate support services, such as obesity management units or primary care resources, to help address these modifiable risk factors and improve long-term disease outcomes.

### Nurse-led education in axSpA patients

AxSpA is characterized by persistent inflammation that frequently results in pain, functional impairment, and reduced quality of life. These physical limitations may contribute to psychological consequences, including anxiety and depressive symptoms, with repercussions on patients’ personal, social, and professional functioning.^54^ Consequently, there is broad agreement that axSpA care should be holistic and multidisciplinary, combining pharmacological treatment with patient education, self-management support, and, when appropriate, psychological counseling or psychotherapy delivered by qualified mental health professionals, with nurses playing a fundamental role in patient and family education.

Nurse-led educational interventions encompass structured activities designed to improve patients’ understanding of their disease across individual, group, and community settings. Rheumatology nurses are particularly well-positioned to support patients in managing axSpA and its associated comorbidities.^54–57^ Core elements of these educational programmes include information and practical training related to diagnostic procedures, pharmacological treatments, physical exercise, pain control strategies, and joint protection. These interventions may also include support for self-monitoring, smoking cessation, vaccination counseling, health literacy, and treatment adherence. Evidence suggests that involvement of specialized rheumatology nurses improves patient satisfaction. In addition, nurse-driven initiatives may support smoking cessation, reduce concerns regarding subcutaneous therapies, assist patients in completing self-assessment tools without influencing treatment choices, and foster self-management and adherence through group-based education. However, the evidence base remains limited, as many studies include mixed populations with inflammatory arthritis or rheumatic diseases rather than axSpA specifically, and outcomes are heterogeneous.

Importantly, nurse-led education should not be regarded as a substitute for specialist psychological care. In patients with significant anxiety, depression, maladaptive coping or marked disease-related distress, referral for formal psychological assessment and, when needed, structured psychological interventions should be considered. Therefore, the role of rheumatology nursing should be understood as complementary: identifying unmet educational and psychosocial needs, reinforcing self-management, and facilitating referral to other professionals, including psychologists or psychiatrists when appropriate.

## Cross-cutting and multidisciplinary aspects

Optimal management of axSpA requires an integrated, patient-centered model of care that extends beyond rheumatology. Given the frequent occurrence of extra-musculoskeletal manifestations, including skin, eye, and gastrointestinal involvement, close collaboration with dermatology, ophthalmology, and gastroenterology is essential to provide coordinated and continuous care. This multidisciplinary approach helps prevent care fragmentation and enables timely recognition and treatment of comorbid conditions.^58^

Within this framework, specialized nursing remains a cornerstone. Educational initiatives delivered either in person or remotely have been associated with improved adherence, greater patient empowerment, and higher satisfaction.^59^ Another key cross-cutting aspect is shared decision-making. Routine incorporation of patient-reported outcomes allows systematic assessment of domains such as fatigue, emotional well-being, and social participation.^60^

Overall, ESPOGUIA 2024 highlights that effective axSpA management requires a comprehensive approach that combines multidisciplinary collaboration, structured nurse-led education, and person-centered care, addressing not only inflammatory activity but also the broader impact of the disease on patients’ daily lives.

## Discussion and future research

Compared with the ASAS-EULAR recommendations,^3^ ESPOGUÍA differs methodologically in that it applies the GRADE framework to assess the quality of evidence and strength of recommendations, whereas ASAS-EULAR relies on the Oxford Centre for Evidence-Based Medicine levels of evidence; in terms of clinical approach, its overall strategy, as both endorse NSAIDs as first-line therapy, escalation to targeted therapy in persistently active disease, attention to extra-musculoskeletal manifestations, and tapering rather than abrupt withdrawal in sustained remission. However, ESPOGUÍA provides more detailed guidance and translates the principles of individualized care more clearly into clinical practice. In contrast to ASAS-EULAR, which recommends choosing a b/tsDMARD according to current practice and extra-musculoskeletal manifestations, ESPOGUÍA places IL-17A, IL-17A/F, and JAK inhibitors as clear post-NSAID options, while also incorporating prognostic factors for treatment response and structural progression into therapeutic decision-making. ESPOGUÍA also gives more detailed practical guidance on treatment optimization, strongly recommending dose-spacing after at least 6 months of low disease activity or remission. Regarding comorbid domains, both documents align in prioritizing monoclonal TNF inhibitors for recurrent uveitis and inflammatory bowel disease, but ESPOGUÍA is more prescriptive by specifically advising against etanercept for uveitis and against IL-17 inhibitors in IBD. Finally, ESPOGUÍA gives greater prominence to lifestyle and multidisciplinary care—including supervised exercise, smoking cessation, weight control, and nurse-led education—reflecting a more practice-oriented and holistic framework. Smoking and obesity were specifically addressed because they represent two of the most prevalent and clinically relevant modifiable comorbidities in axSpA. However, other important conditions, such as diabetes, frailty, and sarcopenia, were not specifically evaluated in this work. This should be considered a limitation and highlights the need for future recommendations and research to adopt a broader approach to comorbidity assessment in axSpA. In addition, although the recommendations emphasize multidisciplinary care, they do not provide detailed guidance on the specific roles and integration of other healthcare professionals, such as cardiologists, nephrologists, physiotherapists/kinesiotherapists, and psychologists, in the management of patients with axSpA. This should also be considered a limitation and underlines the need for future work to better define how multidisciplinary teams can be structured and implemented in routine clinical practice. One aspect not covered in the guidelines is pregnancy and breastfeeding. It was decided to address this issue in conjunction with other conditions in which the same drugs are used, and simply to refer to the recommendations developed specifically by the SER^61^ and, more recently, by EULAR.^62^

Future research in axSpA should prioritize clarifying the boundaries and overlap between axSpA and axial PsA, including comparative studies on disease pathogenesis and imaging-based characterization of vertebral involvement. A deeper understanding of these distinctions would support the development of more precise diagnostic criteria and enhance early recognition of axial disease across the SpA spectrum.

In addition, high-quality studies are needed to evaluate early pharmacological intervention in axSpA, particularly its impact on long-term functional outcomes, structural progression, and quality of life—areas where current evidence remains limited.

Research should also explore optimal long-term treatment strategies in axSpA, including identifying the most appropriate therapy for individual patients and determining which patients may safely benefit from tapering biologic treatment or maintaining remission without continuous therapy. Further studies are required to define the role of different exercise programs in patients across all disease stages, including those with ankylosis or minimal functional limitation. Finally, well-designed education-based interventions delivered by nursing professionals should be developed and evaluated within real-world clinical settings to determine their effectiveness in improving outcomes for individuals living with axSpA.

In addition, emerging digital technologies should become an important focus of future research. These include digital patient-reported outcome collection, remote monitoring tools, wearable devices to capture physical function and activity, and telehealth-based follow-up models. Artificial intelligence and machine learning approaches may also help improve imaging interpretation, identify phenotypic subgroups, predict disease progression and treatment response, and support more personalized care pathways. Such innovations should be carefully developed and validated, with attention to clinical usefulness, interpretability, equity, and implementation in everyday practice.

## Conclusion

This updated edition of ESPOGUÍA provides a comprehensive and evidence-based framework for the management of axial axSpA in Spain, integrating substantial therapeutic advances made in recent years. By systematically reviewing the literature and applying rigorous GRADE methodology, the guideline delivers clear, clinically relevant recommendations that support rheumatologists and multidisciplinary teams in optimizing diagnosis, monitoring, and treatment. The inclusion of newer biologic and targeted synthetic therapies, the evaluation of prognostic factors, guidance on treatment sequencing and tapering, and the careful appraisal of extra-musculoskeletal manifestations reflect the growing complexity of axSpA care and the need for harmonized, patient-centered decision-making. Non-pharmacological strategies such as structured exercise programs, smoking cessation, weight optimization and nurse-led education are also emphasized as essential components of holistic disease management.

ESPOGUÍA further strengthens the importance of collaborative, integrated care involving rheumatology, dermatology, gastroenterology, ophthalmology, physiotherapy, nursing, and primary care. Its recommendations aim not only to reduce unwarranted variability in clinical practice but also to enhance long-term outcomes, quality of life, and patient empowerment. While significant progress has been made, the guideline also highlights important gaps in knowledge—such as the need for robust data on early intervention, radiographic progression, treatment de-escalation, and multidisciplinary educational interventions—thereby guiding future research priorities.

Overall, ESPOGUÍA 2024 stands as a key resource for clinicians and patients, providing practical, evidence-informed guidance to ensure consistent, high-quality care for individuals living with axSpA.

## Supplemental Material

sj-docx-1-tab-10.1177_1759720X261464260 – Supplemental material for Therapeutic management of axial spondyloarthritis: summary of 2024 update of the Spanish clinical practice guideline (ESPOGUIA) Supplemental material, sj-docx-1-tab-10.1177_1759720X261464260 for Therapeutic management of axial spondyloarthritis: summary of 2024 update of the Spanish clinical practice guideline (ESPOGUIA) by Clementina López-Medina, Carlos Montilla-Morales, Mireia Moreno, Manuel Jose Moreno-Ramos, Rubén Queiro, Julio Ramírez, David Díaz-Valle, Agnès Fernández-Clotet, Josep Riera, Petra Díaz-del Campo, Juan D. Cañete and Victoria Navarro-Compán in Therapeutic Advances in Musculoskeletal Disease

## Declarations

**Ethics approval and consent to participate:** Not applicable.

**Consent for publication:** Not applicable.

**Availability of data and materials:** All data and materials used to develop the 2024 ESPOGUÍA recommendations are fully available from the guideline working group. The complete guideline is published by the ESPOGUÍA Working Group, Spanish Society of Rheumatology (SER): Clinical Practice Guideline on the Treatment of Axial Spondyloarthritis and Psoriatic Arthritis, 2024 Update, Madrid, 2024.
