# Supplementary material for: Therapeutic management of axial spondyloarthritis: summary of 2024 update of the Spanish clinical practice guideline (ESPOGUIA)
Source: Ther Adv Musculoskelet Dis. 2026 Aug 1;18:1759720X261464260. doi: 10.1177/1759720X261464260 (PMC13428830; doi:10.1177/1759720X261464260)
Supplement: sj-docx-1-tab-10.1177_1759720X261464260 – Supplemental material for Therapeutic management of axial spondyloarthritis: summary of 2024 update of the Spanish clinical practice guideline (ESPOGUIA) [file sj-docx-1-tab-10.1177_1759720X261464260.docx]

**Supplementary Table 1. Overview of the PICO questions addressed in axial spondyloarthritis.**

| **PICO questions** | |
| --- | --- |
| **Treatment with biologic DMARDs or JAK inhibitors versus placebo** | 1.In patients with axial spondyloarthritis, what is the efficacy of IL-17 inhibitors and JAK inhibitors compared with placebo? |
| **Prognostic factors** | 2. In patients with axial spondyloarthritis, does pharmacological intervention with biologic DMARDs or JAK inhibitors slow the progression of structural damage?  3. In patients with axial spondyloarthritis, what are the prognostic factors for response to treatment with IL-17 inhibitors and JAK inhibitors? |
| **Treatment failure** | 4. In patients with axial spondyloarthritis who have not responded to a TNF inhibitor, is treatment with another TNF inhibitor or another targeted therapy effective? |
| **Treatment optimisation** | 5. In patients with axial spondyloarthritis, can treatment with biologic DMARDs be discontinued or reduced? |
| **Extra-musculoskeletal manifestations** | 6. In patients with axial spondyloarthritis, what is the efficacy of biologic DMARDs and targeted synthetic DMARDs in extra-musculoskeletal manifestations (uveitis, psoriasis, and inflammatory bowel disease)? |
| **Exercise** | 7. In patients with axial spondyloarthritis, which type of exercise programme is most effective in improving clinical and functional outcomes? |
| **Smoking and obesity** | 8. In patients with axial spondyloarthritis, do smoking and obesity worsen disease activity, radiographic progression, and treatment response? |
| DMARDs: Disease modifying anti-rheumatic drugs. JAK: Janus kinases. | |
